# Copy Number Variants Account for a Tiny Fraction of Undiagnosed Myopathic Patients

**DOI:** 10.3390/genes9110524

**Published:** 2018-10-26

**Authors:** Teresa Giugliano, Marco Savarese, Arcomaria Garofalo, Esther Picillo, Chiara Fiorillo, Adele D’Amico, Lorenzo Maggi, Lucia Ruggiero, Liliana Vercelli, Francesca Magri, Fabiana Fattori, Annalaura Torella, Manuela Ergoli, Anna Rubegni, Marina Fanin, Olimpia Musumeci, Jan De Bleecker, Lorenzo Peverelli, Maurizio Moggio, Eugenio Mercuri, Antonio Toscano, Marina Mora, Lucio Santoro, Tiziana Mongini, Enrico Bertini, Claudio Bruno, Carlo Minetti, Giacomo Pietro Comi, Filippo Maria Santorelli, Corrado Angelini, Luisa Politano, Giulio Piluso, Vincenzo Nigro

**Affiliations:** 1 Dipartimento di Medicina di Precisione, Università degli Studi della Campania “Luigi Vanvitelli”, 80138 Napoli, Italy; teresa.g86@gmail.com (T.G.); marco.savarese@helsinki.fi (M.S.); garofalo.arca@gmail.com (A.G.); annalaura.torella@gmail.com (A.T.); giulio.piluso@unina2.it (G.P.); vinnigro@gmail.com (V.N.); 2 Telethon Institute of Genetics and Medicine, 80078 Pozzuoli, Italy; 3 Folkhälsan Research Center, Medicum, University of Helsinki, FI-00290 Helsinki, Finland; 4 Cardiomiologia e Genetica Medica, Dipartimento di Medicina Sperimentale, Università degli Studi della Campania “Luigi Vanvitelli”, 80138 Napoli, Italy; estherstar@libero.it (E.P.); manuergoli@libero.it (M.E.); luisa.politano@unicampania.it (L.P.); 5 U.O.C. Neurologia Pediatrica e Malattie Muscolari, IRCCS Istituto Giannina Gaslini, 16147 Genova, Italy; chi.fiorillo@gmail.com (C.F.); claudio2246@gmail.com (C.B.); minettic@unige.it (C.M.); 6 Unit of Neuromuscular and Neurodegenerative Disorders, Laboratory of Molecular Medicine, “Bambino Gesù” Children’s Hospital, IRCCS, 00146 Roma, Italy; adeledamico@hotmail.com (A.D.); fabianafattori79@gmail.com (F.F.); ebertini@gmail.com (E.B.); 7 Neuromuscular Diseases and Neuroimmunology Unit, Istituto Besta, 20133 Milano, Italy; lorenzo.maggi@istituto-besta.it (L.M.); Marina.Mora@istituto-besta.it (M.M.); 8 Dipartimento di Neuroscienze e Scienze Riproduttive ed Odontostomatologiche, Università degli Studi di Napoli “Federico II”, 80131 Napoli, Italy; ruggilucia@gmail.com (L.R.); lucio.santoro@unina.it (L.S.); 9 S.S. Malattie Neuromuscolari, Università degli Studi di Torino, 10124 Torino, Italy; lilianavercelli@hotmail.com (L.V.); tmongini@gmail.com (T.M.); 10 Centro Dino Ferrari, Dipartimento di Fisiopatologia Medico-Chirurgica e dei Trapianti, Università degli Studi di Milano, Fondazione IRCCS Ca’ Granda, Ospedale Maggiore Policlinico, 20122 Milano, Italy; francescam.magri@gmail.com (F.M.); giacomo.comi@unimi.it (G.P.C.); 11 Medicina Molecolare, IRCCS Fondazione Stella Maris, 56128 Pisa, Italy; anna.rubegni@gmail.com (A.R.); filippo3364@gmail.com (F.M.S.); 12 Fondazione Hospital S.Camillo IRCCS, 30126 Venezia, Italy; marina.fanin@unipd.it (M.F.); corrado.angelini@unipd.it (C.A.); 13 Department of Clinical and Experimental Medicine, University of Messina, 98122 Messina, Italy; omusumeci@unime.it (O.M.); atoscano@unime.it (A.T.); 14 Department of Neurology, Ghent University Hospital, 9000 Ghent, Belgium; Jan.DeBleecker@ugent.be; 15 Neuromuscular and Rare Disease Unit, Dipartimento di Neuroscienze, Università degli Studi di Milano, Fondazione IRCCS Ca’ Granda, Ospedale Maggiore Policlinico, 20122 Milano, Italy; lorenzo.peverelli@policlinico.mi.it (L.P.); maurizio.moggio@unimi.it (M.M.); 16 Istituto di Neurologia, Università Cattolica del Sacro Cuore, Fondazione Policlinico Universitario “A. Gemelli”, 00168 Roma, Italy; eumercuri@gmail.com

**Keywords:** copy number variants, skeletal muscle disorders, next-generation sequencing, variants of uncertain significance

## Abstract

Next-generation sequencing (NGS) technologies have led to an increase in the diagnosis of heterogeneous genetic conditions. However, over 50% of patients with a genetically inherited disease are still without a diagnosis. In these cases, different hypotheses are usually postulated, including variants in novel genes or elusive mutations. Although the impact of copy number variants (CNVs) in neuromuscular disorders has been largely ignored to date, missed CNVs are predicted to have a major role in disease causation as some very large genes, such as the dystrophin gene, have prone-to-deletion regions. Since muscle tissues express several large disease genes, the presence of elusive CNVs needs to be comprehensively assessed following an accurate and systematic approach. In this multicenter cohort study, we analyzed 234 undiagnosed myopathy patients using a custom array comparative genomic hybridization (CGH) that covers all muscle disease genes at high resolution. Twenty-two patients (9.4%) showed non-polymorphic CNVs. In 12 patients (5.1%), the identified CNVs were considered responsible for the observed phenotype. An additional ten patients (4.3%) presented candidate CNVs not yet proven to be causative. Our study indicates that deletions and duplications may account for 5–9% of genetically unsolved patients. This strongly suggests that other mechanisms of disease are yet to be discovered.

## 1. Introduction

Prior to the advent of next-generation sequencing (NGS), high phenotypic overlapping and lack of pathognomonic signs in neuromuscular disorders (NMDs) made clinical diagnosis difficult and molecular confirmation extremely complex and challenging [1]. In the NGS era, different genetic approaches have been described in literature [2,3] with a detection rate of single nucleotide variants or small ins/dels ranging between 40% and 60% [4].

Patients remaining undiagnosed may harbor mutations in unknown genes, multifactorial or polygenic conditions, or elusive variants such as deep intronic mutations, variants in regulatory elements, trinucleotide repeat expansions and copy number variants (CNVs) [5].

CNVs are defined as genomic deletions and duplications of 50 base pairs (bp) or longer [6] that may account for 5–10% of elusive mutations in the human genome [7]. To date, few custom array comparative genomic hybridization (CGH) studies on muscular genes have been described [8,9,10]. Recently, CNVs were detected from target sequencing, whole exome, or genome data by applying different computational algorithms [11,12]. Despite the use of bioinformatics tools, array CGH remains the gold standard for detection of exon CNVs [13].

Previous reports suggested that the frequency of CNVs in myopathy patients is around 4–10% [9]. However, these studies mainly provided a technical validation of array CGH platforms [8,9], with a limited number of genes [10] and/or patients tested [9]. In this study, we enrolled 234 patients who had remained undiagnosed after an extensive molecular investigation including Sanger sequencing of candidate genes and MotorPlex, a targeted NGS panel designed to detect single-base substitutions or small ins/dels [14].

We analyzed these genetically undiagnosed patients by Motor Chip, a custom array CGH for the detection of deletions and duplications in 425 neuromuscular genes [9], and identified causative and potential disease-causing CNVs in 22 patients.

## 2. Materials and Methods

### 2.1. Patients Recruited

We collected DNA samples from 504 patients presenting with clinical signs of limb-girdle muscular dystrophies, congenital myopathies and other conditions affecting the muscles including isolated hyperCKemia. All samples were analyzed by a targeted NGS tool, named MotorPlex [14,15], to identify single nucleotide changes as well as small ins/dels. Based on DNA availability, 234 out of 286 myopathic patients who had remained undiagnosed after this preliminary NGS study were recruited to look for CNVs. The patients involved in the project GUP11006 had already provided written informed consent at the time of blood collection and the Ethics Committee of the University of Campania, Naples, Italy approved the extension of the project [14]. Genomic DNA from leukocytes of peripheral blood was isolated using standard operating procedures established by the EuroBioBank network. DNA quality and quantity were assessed using spectrophotometric (Nanodrop ND 1000, Thermo Scientific Inc., Rockford, IL, USA) and fluorometry-based (Qubit 2.0 Fluorometer, Life Technologies, Carlsbad, CA, USA) methods.

### 2.2. Array CGH and Pair Analysis

All the undiagnosed patients were enrolled for this study immediately after analysis of NGS results in 2015. At that time, few bioinformatics tools were available for CNV analysis on whole exome data, but none had been optimized for use on data from targeted NGS. We used the most user-friendly tool, SureCall (Agilent Technologies, Santa Clara, CA, USA) and tested its ability to identify CNVs from 50 targeted NGS samples. The SureCall algorithm uses pair analysis (sample vs. reference), comparing the per base read depth coverage of each target interval for sample and reference BAM files. Aberration intervals were predicted by the log_2_ ratio of depth coverage of the sample to the reference.

A custom array CGH named Motor Chip, able to investigate more than 400 genes related to neuromuscular disorders with an exonic resolution, was then used for the detection and characterization of CNVs [9]. Labeling and hybridization were performed using SureTag labeling kit (Agilent Technologies) according to the manufacturer’s specifications. Scanned array images were analyzed by Cytogenomics v4 (Agilent Technologies). After performing a quality control test, duplications and deletions were identified using the Aberration Detection Method 2 (ADM-2) algorithm. At least three target probes with changes in the number of copies were required for a CNV call. Deletions and duplications corresponding to well-known copy number polymorphisms were filtered off [16]. Variants not known to be pathogenic or of doubtful significance were compared with the Database of Genomic Variants [17], DECIPHER (https://decipher.sanger.ac.uk/), and ExAC Browser (http://exac.broadinstitute.org/) to facilitate their interpretation. Data reported here are submitting to ClinVar [18] and Leiden [19].

### 2.3. Validation Experiments

#### 2.3.1. Real-Time PCR

All identified CNVs were further confirmed by real-time PCR using the Bio-Rad CFX96 system (Bio-Rad, Pleasanton, CA, USA). Specific primers (available from the authors on request) able to investigate the deleted or duplicated regions were designed using the Primer 3 webtool. The 20 µL reaction contained 10 µL 2X FastStart universal SYBR Green Master (Bio-Rad), 10 µM of each primer, and 10 ng of genomic DNA as template. The following thermal conditions were used: 10 min of preheating at 95 °C followed by 45 cycles of 15 s at 95 °C, 30 s at 62 °C and an extension of 68 °C for 30 s. Quantitative analysis was performed by CFX Manager (Bio-Rad). The identified variants are reported based on the following coding DNA reference sequence, as suggested by the Human Genome Variation Society (HGVS) recommendations: *DMD* (NM_004006.2), *LAMA2* (NM_000426.3), *SGCD* (NM_000337.5), *SGCB* (NM_000232.4), *SGCG* (NM_000231.2), *SPAST* (NM_014946.3).

#### 2.3.2. MLPA Analysis for *DMD* and *SPAST* Genes

Complete or partial deletions/duplications involving *DMD* and *SPAST* genes were confirmed by Multiplex ligation-dependent probe amplification (MLPA) using the SALSA MLPA P034 and P035 DMD kits and P165 SPAST kit (MRC-Holland, Amsterdam, The Netherlands), according to the manufacturer’s recommendations. MLPA data analysis was performed with the Coffalyser.net package (MRC-Holland).

#### 2.3.3. cDNA Analysis

To further characterize the duplications, a detailed analysis of the transcript was conducted. RNA was isolated from leukocytes of peripheral blood or from muscular biopsies, according to standard procedures. A specific coding sequence demonstrating the predicted tandem duplications was then amplified and bidirectionally sequenced on an ABI 3130xL automatic DNA sequencer (Applied Biosystems, Foster City, CA, USA).

#### 2.3.4. Histological Studies and Western Blot Analysis

Histological and histochemical examinations in muscle biopsies were carried out following standard procedures [20]. Western blotting of muscle biopsy samples was performed according to standard methods [21].

## 3. Results

**Figure 1:**
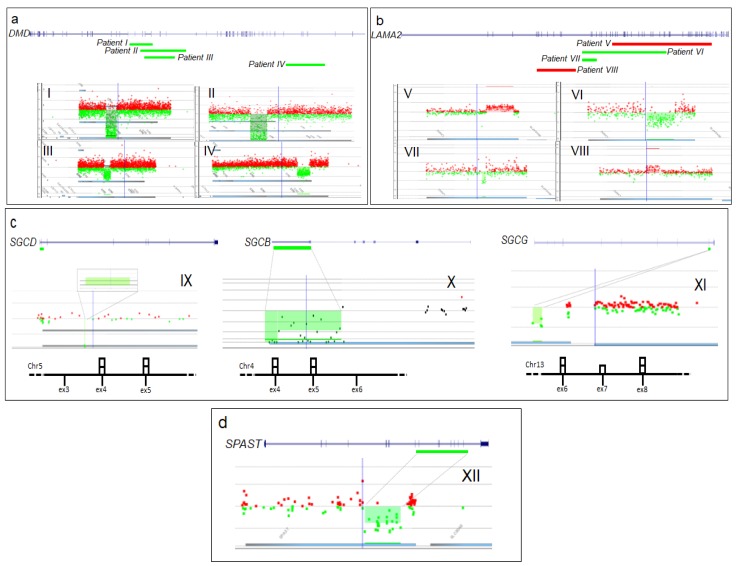
Graphic view of 12 causative copy number variants (CNVs). Gene representation and array CGH panels showing: (**a**) 4 *DMD* deletions (patients I–IV); (**b**) 2 deletions and 2 duplications in *LAMA2* (patients V–VIII); (**c**) 3 deletions involving sarcoglycan genes (patients IX–XI); (**d**) *SPAST* gene deletion in patient XII. Green bars indicate deletions and red bars indicate duplications. The results of genomic quantification by real-time PCR are schematically represented (bottom of **c**). The number of each coding exon along gene is surmounted by a square corresponding to the number of detected copies.

After NGS analysis, 50 undiagnosed patients were initially tested for CNVs by SureCall pair analysis, which confidently identified on average eight CNV calls per sample. Motor Chip, performed to validate the identified CNVs, showed that SureCall pair analysis achieved a detection rate of about 40–60%. Because of the high number of CNV calls with low sensitivity and the lack of accuracy in CNV breakpoint mapping -with the SureCall pair analysis, we used Motor Chip to further test the remaining undiagnosed patients. Motor Chip analysis identified a causative deletion or duplication in genes responsible for the observed phenotype in 12 out of 234 patients (5.1%) (Figure 1). Molecular findings and clinical data of patients carrying the identified CNVs are summarized in Table 1.

## 4. Causative CNVs

### 4.1. CNVs in Dystrophin Gene

Previously, described dystrophin gene (*DMD*-MIM 300377) deletions [22] were identified in four patients (two men and two women) presenting with proximal weakness and/or isolated serum CK levels 10 times over the maximum normal range (patients I–IV). The deletion of exons 3–7 in patient IV occurred de novo. In patient III the deletion of exons 45–48 was inherited from her affected father, who had remained undiagnosed due to a very mild phenotype and a very low increase in CK values.

### 4.2. CNVs in Laminin-2 Gene

**Figure 2:**
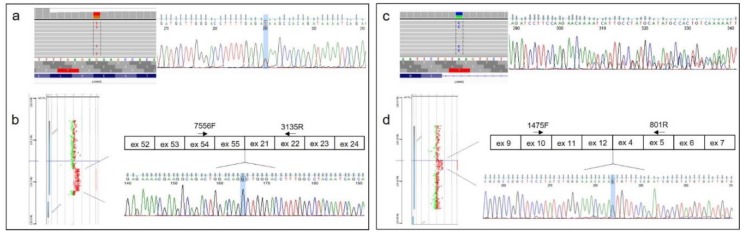
Molecular characterization of *LAMA2* variants in patients V and VIII. Single nucleotide variants in *LAMA2* occurred in compound heterozygosity with a duplication. (**a**) Integrative Genomics Viewer (IGV) [27] visualization and Sanger confirmation of c.5374G>T (patient V); (**b**) Characterization of exon 21–55 duplication on RNA (patient V). (**c**) The splice variant c.6429+3A>C characterized on muscle transcript, leads to skipping of exons 44–45 (patient VIII); (**d**) Fine mapping of the tandem duplication on muscle RNA transcript (patient VIII).

In four patients, laminin-2 gene (*LAMA2*-MIM 156224) CNVs were detected in the presence of a previously identified heterozygous single nucleotide mutation by MotorPlex. In particular, patient V carried an unreported nonsense mutation (c.5374G>T, p. Glu1792*) and a 190.6 kb intragenic duplication. A further molecular characterization of the LAMA2 transcript, isolated from leukocytes, showed a tandem, in-frame duplication involving exons 21–55 of the gene (Figure 2a,b). A merosin deficiency was confirmed by Western blotting of skeletal muscle. Patient VI harbored a previously unreported missense variant in exon 47 of *LAMA2* gene (c.6599G>A, p.Arg2200His) and a known heterozygous in-frame deletion of exons 13–37 [9] in the same gene. Array CGH identified a novel heterozygous *LAMA2* deletion (exons 13–14) generating a frameshift of open reading frame in patient VII, affected by a congenital myopathy. In the same patient, MotorPlex had identified a heterozygous mutation affecting the canonical splice site (c.4312-1G>A) of exon 29.

Similarly, patient VIII harbored a previously undescribed intronic variant (c.6429+3A>C) as well as a 75 kb intragenic duplication (exons 4–12). Muscular RNA analysis identified two transcripts (Figure 2c,d): one transcript with skipping of exons 44 and 45 leading to the loss of reading frame (due to the intronic splicing variant) and a second transcript showing an in-frame duplication of 1,386 nucleotides, predicted to produce a protein longer by 462 amino acids. However, we were unable to perform a Western bot analysis as additional tissue was not available.

### 4.3. CNVs in Sarcoglycan Genes

In patient IX, we found a previously unreported homozygous deletion of the first coding exon of δ-sarcoglycan gene (*SGCD*-MIM 601411). His unaffected parents were heterozygous for the observed CNV. Similarly, the strong clinical suspicion for patient X and the lack of a causative variant after NGS analysis prompted us to check the coverage for sarcoglycan genes. NGS data coverage showed the absence of reads in the last exon of beta-sarcoglycan gene (*SGCB*-MIM 600900) suggesting a deletion. Motor Chip analysis defined the deletion at the last 12 codons in exon 6 and the 3’ UTR (untranslated region) [23]. We also detected a reported heterozygous deletion spanning exon 7 of gamma-sarcoglycan gene (*SGCG*-MIM 608896) on the maternal allele of patient XI [24]. In the same patient, NGS analysis had previously identified an in-frame 3 nucleotide deletion (c.124_126del), listed in the LOVD database [19], on the father’s allele in exon 2 of *SGCG* gene.

### 4.4. Deletion in Spastin Gene

We found a reported in-frame heterozygous deletion of exons 10–16 [25] of spastin gene (*SPAST*-MIM 604277) in patient XII, who presented with fatigue and hyposthenia since adolescence. A large segregation analysis showed the presence of the deletion in her three affected relatives (III-1, III-8, IV-3; Supplementary Figure S1) and in her asymptomatic sister (III-7; Supplementary Figure S1). Three unaffected family members (III-3, III-4, III-6; Supplementary Figure S1) were negative for the observed deletion, while genetic testing was not performed in the clinically affected patients II-3 and IV-1, who refused DNA investigations.

We identified variants of uncertain significance (VUS) in ten patients (4.3%) (Table 2). VUS are genomic variants not previously reported in normal individuals and with insufficient information about their clinical significance [26]. As required by the American College of Medical Genetics and Genomics guidelines, an exhaustive characterization will be needed to clarify their clinical meaning.

## 5. Discussion

### 5.1. Role of CNVs in Skeletal Muscle Disorders

Whole exome and targeted sequencing have proven to be robust and cost-efficient diagnostic tools in heterogeneous diseases, increasing the detection rate of disease-causing variants compared to the traditional gene-by-gene approach [28]. However, over 50% of undiagnosed patients affected by nonspecific skeletal muscle disorders do not receive any molecular diagnosis using NGS strategies [14].

Taking advantage of a large cohort of myopathy patients previously screened with a gene panel assay, we designed a study aiming to verify the presence of CNVs in unsolved patients [14]. An extensive screening of 234 myopathy patients by Motor Chip identified 22 CNVs. A causative deletion or duplication was found in 12 out of the 234 patients (5.1%).

The identification of deletions or duplications in *DMD* gene causing Becker muscular dystrophy (BMD) was not unexpected, and only confirmed the clinical overlap between BMD and limb-girdle muscular dystrophy [29]. In total, 2.2% of the original 504 patients recruited to the study were diagnosed with BMD, including those carrying the 7 single nucleotide variants (small indels, nonsense and splice site variants) identified by MotorPlex [14].

The presence of female carriers as isolated cases in the absence of affected males in the family (case IV) is noteworthy. The diagnosis of a Duchenne muscular dystrophy or BMD manifesting carrier should be suspected in a female presenting with limb-girdle weakness, despite a negative family history [30]. As previously reported for X-Linked myotubular myopathy [31,32], the extensive use of genome-wide tools in a diagnostic setting will identify an increasing number of BMD manifesting carriers, probably due to skewed X-chromosome inactivation [33]. Their identification will be crucial for appropriate genetic counseling and correct prenatal diagnosis.

Several *LAMA2* intragenic deletions and duplications were previously reported [34]. In our cohort, we found a CNV in four out of 15 patients (27%) harboring a rare (minor allele frequency < 1%) single nucleotide variant in *LAMA2*. Interestingly, *LAMA2* CNVs in compound heterozygosity with a protein truncating variant (PTV) were observed in three patients. Therefore, in the presence of a heterozygous PTV, a CNV affecting the second allele should always be suspected.

CNVs in sarcoglycan genes are generally considered a rare event. However, our results, as well as previous data [9], suggest that their incidence has likely been underestimated. As in the case of *LAMA2*, screening for exonic deletions/duplications in sarcoglycan genes is strongly recommended in patients carrying a single heterozygous mutation, above all if a reduction of sarcoglycan proteins is observed [23].

Deletions in *SPAST* gene are common in hereditary spastic paraplegia [35]. However, the high phenotypic variability of the disease makes clinical evaluation and molecular characterization challenging, especially when proximal muscle weakness in the limbs and minimal neurological signs are present [36], as in patient XII. In this case, identification of the *SPAST* deletion provided the molecular diagnosis in her family, increasing the number of described cases. As previously reported, the presence of an asymptomatic carrier of a spastin deletion may be considered a reduced penetrance of the disorder [36].

Retrospectively, we performed a SureCall analysis using 10 out of 12 causative CNVs as positive controls. Although in four samples (II, VI, VIII, IX) we identified the respective deletion or duplication previously found with array CGH, breakpoints were not correctly defined. Unlike MotorPlex, which provides exon level coverage, Motor Chip design comprises probes covering the intronic region of neuromuscular genes, yielding a better definition of CNV breakpoints.

### 5.2. Variants of Uncertain Significance

We identified non-recurrent rare CNVs, which we interpreted as VUS, in ten patients (4.3%) including heterozygous deletions involving genes responsible for autosomal recessive muscle disorders (*ETFB*-patient XIII; *MLYCD*-patient XIV). Despite our exhaustive genetic investigation, including an NGS strategy and a CNV assessment, the presence of further elusive variants (such as deep intronic variants or small repeat changes) in these genes cannot be excluded.

Two interesting heterozygous deletions were found in genes (*MYPN-*patient XV and *CSRP3-*patient XVI) associated with a dominant cardiomyopathy. Specifically, an *MYPN* deletion was found in patient XV presenting with hypertrophic cardiomyopathy and centronuclear myopathy. Biallelic mutations of *MYPN* are associated with a slowly progressive nemaline myopathy [37] and a recessive cap myopathy [38]. However, no other single nucleotide variants in *MYPN* were identified in this patient. Still more complex is the interpretation of the *CSRP3* deletion identified in patient XVI, who presented with dilated cardiomyopathy and muscle weakness. To date, no *CSRP3* mutations have been associated with a skeletal muscle phenotype. However, no other variants in myopathy disease genes included in our Motor Chip and MotorPlex panels explain the observed phenotype in these two cases. Although *MYPN* and *CSRP3* may have a role in the observed cardiomyopathies, the primary genetic cause of the skeletal muscle disorder in these patients remains to be identified.

A duplication of the 15q11.2 region was found in patient XVII affected by congenital arthrogryposis. The patient recently obtained a clinical diagnosis of Nail Patella syndrome, confirmed by the identification of a de novo variant in *LMX1B* gene. However, the role of the identified duplication is still unclear since CNVs in the 15q11.2 region are normally associated with neurodevelopmental disorders [39,40].

Segregation studies in only two cases, patient XVIII and patient XIX excluded the primary disease role of a heterozygous deletion of *SPG11* gene and Xp22.13-p22.12, respectively.

In contrast, the *G6PC* deletion detected in patient XX and the *SEPN1* duplication found in patient XXI are not sufficient to explain the observed clinical phenotype in the absence of comprehensive biochemical and molecular evidence. Lastly, a heterozygous deletion of 1q23.3-24.2 was identified in patient XXII presenting with core myopathy. As this rearrangement involves five disease genes not previously associated with core myopathy, its role remains unclear.

In sum, although these VUS may not act as primary disease drivers, we cannot exclude the possibility that some may play a role as modifiers, contributing to the observed phenotype. However, the absence of functional assays and the lack of comparisons with similar cases are a limitation in the clinical interpretation of these rearrangements.

## 6. Conclusions

Here, we report the results observed in the largest cohort of patients with a skeletal muscle disorder analyzed to date by array CGH. In line with data reported in literature, our findings confirm that deletions and duplications are present in 5–9% of patients affected by a skeletal muscle disorder without a molecular diagnosis. However, further extensive studies will be needed to clarify the role of CNVs in about half of these cases. The addition of data (including frequency) on CNVs to the ExAC database may assist in the clinical interpretation of observed rearrangements [41]. However, as evidenced here as well as by others, the integration of NGS results with biochemical, histological and Western blotting findings remains crucial for a correct clinical and diagnostic evaluation [23,42].

Although NGS panels are extensively applied in clinical settings for the detection of single nucleotide variants or small ins/dels, identification of deletions or duplications of whole exons, particularly single-exon CNVs, has proved problematic [43].Improvements to tools identifying CNVs from NGS data are regularly reported [11,12]. Since several algorithms used to detect CNVs from NGS data [11] are now available, the exclusive adoption of SureCall for the identification of CNVs from NGS data may represent a limitation of this study. We are aware that more advanced algorithms may likely have higher sensitivity and specificity. However, their use still generates a high number of false positives, and their readouts still require validation by other methods such as array CGH or MLPA for further mapping of breakpoints. Array CGH remains the gold standard method for CNV detection and analysis, especially in diagnostics, as it is a well-established and validated strategy. Noteworthy, our study suggests that Sanger sequencing is still the only reliable method for determining exact breakpoints at base-pair level, especially for duplications.

Since muscle tissues express several very large disease genes, some of which are prone to deletions or duplications, further assessment of possible CNVs is strongly advised in neuromuscular diagnostic settings. Finally, the use of whole genome sequencing or single-molecule long-read sequencing will help extend the search to other elusive variants not detectable by targeted or whole exome sequencing and CNV mapping [44].

## Figures and Tables

**Table 1:** Causative copy number variants.

ID	Onset	Symptoms Referred	Muscle Weakness (Distribution)	Serum CK	EMG	Biopsy Findings	Other	Gene	Allele 1	Allele 2	Reading Frame
I	childhood	muscle weakness	lower limbs	>20X	myopathic	dystrophic features	RF:2950 mL (75%)	*DMD*	hem del: exons 45–51 (mat)	-	in frame
II	childhood	muscle weakness	lower limbs	>20X	myopathic	dystrophic features	n.a.	*DMD*	hem del: exons 45–49 (U)	-	in frame
III	-	hyperCKemia	asymptomatic	>20X	n.a.	myopathic features	RF:2550 mL (92%)	*DMD*	het del: exons 45–48 (pat)	-	in frame
IV	adult	muscle weakness	lower limbs	>20X	n.a.	dystrophic features	RF:2720 mL (104%)	*DMD*	het del: exons 3–7 (de novo)	-	out of frame
V	juvenile	epilepsy	mild scapula winging, no muscle weakness	>20X	n.a.	dystrophic features with partial merosin deficiency (both 80 and 300 kD)	n.a.	*LAMA2*	dup: exons 21–55 (pat)	c.5374G>T, p. Glu1792* (ex 37) (mat)	in frame
VI	congenital	psychomotor delay	normal	normal	n.a.	n.a.	autism	*LAMA2*	del: exons 13–37 (pat)	c.6599G>A, p. Arg2200His (ex 47) (mat)	in frame
VII	juvenile	distal weakness	slight bilateral scapula winging, positive Gowers sign, mild waddling gait, slight atrophy of the right more than left upper > lower limbs	6–10X	myopathic	n.a.	mild pectus excavatum	*LAMA2*	del: exons 13–14 (mat)	c.4312-1G>A p.? (ex 29) (pat)	out of frame
VIII	juvenile	difficulties in walking	lower limbs	>10X	n.a.	n.a.	RF:2400 mL (72%)	*LAMA2*	dup: exons 4–12 (pat)	c.6429+3A>C, p. Iso2144Glnfs7* (ex 45) (mat)	in frame
IX	juvenile	muscle weakness	proximal, lower > upper limbs	>20X	myopathic	dystrophic features	RF:2720 mL (78%)	*SGCD*	del: first coding exon (pat)	del: first coding exon (mat)	in frame
X	juvenile	muscle weakness	proximal	>20X	myopathic	dystrophic features	n.a.	*SGCB*	del: last 12 codons in exon 6 and 3’ UTR (U)	del: last 12 codons in exon 6 and 3′ UTR (U)	in frame
XI	childhood	difficulties in walking	proximal four limbs	>20X	n.a.	dystrophic features	RF:3380 mL (67%) and dilated cardiomyopathy	*SGCG*	del: exon 7 (mat)	c.124_126delTTC p. Leu41del (ex2) (pat)	out of frame
XII	juvenile	hyposthenia, fatigue	proximal in the lower limbs	normal	mixed	n.a.	n.a.	*SPAST*	del: exons 10–16	-	in frame

Abbreviations: n.a. = not available, CK = creatine kinase, EMG = electromyography, RF = respiratory function, mat = maternal, pat = paternal, U = unknown, het = heterozygous; hem = hemizygous, UTR = untranslated region.

**Table 2:** Variants of uncertain significance.

ID	Onset	Symptoms Referred	Muscle Weakness (Distribution)	Serum CK	EMG	Biopsy Findings	Other	CNV (Min Interval) hg19	Description	Reading Frame
XIII	adult	difficulties in walking	lower limbs	6X	n.a.	n.a.	RF:4330 mL (124%) and atrial septum defect	chr19:51857476-51871484	het del: *ETFB* exons 1–2	n.a.
XIV	juvenile	shoulder and pelvic girdle weakness	severe proximal muscle weakness, wheelchair bound	3X	myopathic	dystrophic features	mild respiratory insufficiency	chr16:83342046-83949780	het del: *CDH13*, *HSBP1* and *MLYCD*	n.a.
XV	juvenile	proximal weakness	weakness in posterior muscles and quadriceps and distal in lower limbs	N/10X	myopathic	aspecific, internal nuclei	slight cardiac hypertrophy	chr10:69898720-69909802	het del: *MYPN* exons 3–5	out of frame
XVI	young adult	limb-girdle weakness	proximal and axial	15X	n.a.	dystrophic features, neurogenic and myofibrillar damage, partial αDG reduction	dilated cardiomyopathy	chr11:18536283-19213867	het del: *CSRP3* exons 4–7 and 3′UTR	n.a.
XVII	congenital	congenital arthrogryposis	none	N	n.a.	fiber type dystroportion	-	chr15:22756504-23088787	dup: *TUBGCP5*, *CYFIP1*, *NIPA2* and *NIPA1*	n.a.
XVIII	juvenile	distal weakness	proximal and distal	N	myopathic	dystrophic features, central nuclei, increased connectival tissue and rare vacuoles	-	chr15:44862731-44900870	het del: *SPG11* exons 18–32	in frame
XIX	childhood	skin problem	proximal and distal, not walking	n.a.	n.a.	n.a.	-	chrX:18910408-30489847	het del: 34 genes*	n.a.
XX	adult	hyperCKemia	proximal	7X	normal	myopathic features	-	chr17:41050890-41053142	het del: *G6PC* exon 1 (pat)	n.a.
XXI	young adult	lower limb distal weakness	distal involvement, with steppage, mild-to moderate weakness of trapezius and iliopsoas, mild bulbar involvement, minima facial weakness	N	mixed	core myopathy, desmin accumulation	atrial fibrillation and flutter, bilateral atrial dilatation, right branch block	chr1:26140297-26140584	dup: *SEPN1* exons 11–12	n.a.
XXII	juvenile	muscle weakness	n.a.	15X	n.a.	core myopathy	-	chr1:164682483-168354339	het del: 21 genes *	n.a.

Abbreviations: n.a. = not available, CK = creatine kinase, EMG = electromyography, RF = respiratory function, pat = paternal, DG = dystroglycan, N = normal, het = heterozygous; del = deletion; dup = duplication. * list of genes is available upon request.

## Supplementary Materials

The following are available online at http://www.mdpi.com/2073-4425/9/11/524/s1, Figure S1: Pedigree of family with the *SPAST* deletion. Female III-5 in the pedigree corresponds to patient XII, harboring deletion of exons 10–16 of *SPAST* gene.
