# Supplementary figures and images for: Copy Number Variants Account for a Tiny Fraction of Undiagnosed Myopathic Patients
Source: Genes (Basel). 2018 Oct 26;9(11):524. doi: 10.3390/genes9110524 (PMC6267442; doi:10.3390/genes9110524)

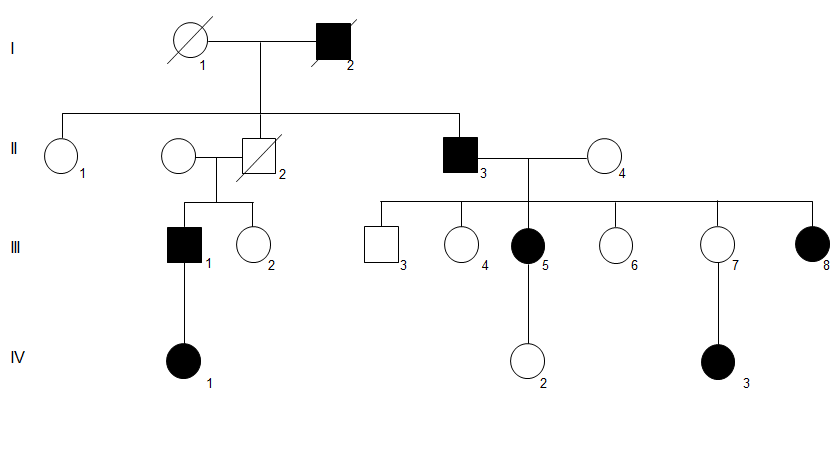

Supplement: Supplementary file 1 [file genes-09-00524-s001.zip › genes-364940-SI.tif]
